# Hydroxyapatite-Based Natural Biopolymer Composite for Tissue Regeneration

**DOI:** 10.3390/ma17164117

**Published:** 2024-08-20

**Authors:** Wasan Alkaron, Alaa Almansoori, Katalin Balázsi, Csaba Balázsi

**Affiliations:** 1 Institute for Technical Physics and Materials Science, HUN-REN Centre for Energy Research, Konkoly-Thege Miklós Str. 29-33, 1121 Budapest, Hungary; alaa.almansoori@ek.hun-ren.hu (A.A.); balazsi.katalin@ek.hun-ren.hu (K.B.); 2 Doctoral School of Materials Science and Technologies, Óbuda University, Bécsi Str. 96/B, 1030 Budapest, Hungary; 3 Technical Institute of Basra, Southern Technical University, Basra 61001, Iraq

**Keywords:** hydroxyapatite, biopolymers, composite, tissue regeneration

## Abstract

Hydroxyapatite (HAp) polymer composites have gained significant attention due to their applications in bone regeneration and tooth implants. This review examines the synthesis, properties, and applications of Hap, highlighting various manufacturing methods, including wet, dry, hydrothermal, and sol–gel processes. The properties of HAp are influenced by precursor materials and are commonly obtained from natural calcium-rich sources like eggshells, seashells, and fish scales. Composite materials, such as cellulose–hydroxyapatite and gelatin–hydroxyapatite, exhibit promising strength and biocompatibility for bone and tissue replacement. Metallic implants and scaffolds enhance stability, including well-known titanium-based and stainless steel-based implants and ceramic body implants. Biopolymers, like chitosan and alginate, combined with Hap, offer chemical stability and strength for tissue engineering. Collagen, fibrin, and gelatin play crucial roles in mimicking natural bone composition. Various synthesis methods like sol–gel, hydrothermal, and solution casting produce HAp crystals, with potential applications in bone repair and regeneration. Additionally, the use of biowaste materials, like eggshells and snails or seashells, not only supports sustainable HAp production but also reduces environmental impact. This review emphasizes the significance of understanding the properties of calcium–phosphate (Ca-P) compounds and processing methods for scaffold generation, highlighting novel characteristics and mechanisms of biomaterials in bone healing. Comparative studies of these methods in specific applications underscore the versatility and potential of HAp composites in biomedical engineering. Overall, HAp composites offer promising solutions for improving patient outcomes in bone replacement and tissue engineering and advancing medical practices.

## 1. Introduction

In the early 2000s, there was a prevalent interest in studying materials possessing bio-related properties and compatibility with the human body [1]. Hydroxyapatite Ca_10_(PO_4_)_6_(OH)_2_ (HAp), a ceramic material resembling bone composition, gained significant attention due to its synthesis in similar structures. This has led to the development of a new class of materials known as hydroxyapatite composites, formed by combining calcium compounds with polymers. These composites exhibit corrosion resistance, good strength-to-weight ratios, and organic nature [2,3,4]. The crystal structure, microstructure, and mechanical behavior of HAp play crucial roles in its manufacturing process to ensure similarity to natural bone. Various methods, including solution casting, hydrothermal, sol–gel, and wet methods, are employed for HAp preparation [5,6,7,8]. The preparation methods also influence the final properties of HAp and HAp composites [6]. Simple reactions, such as calcium hydroxide reacting with phosphoric acid, can also yield hydroxyapatite [9].

The human bones are composed of 70% inorganic components (HAp) and 30% organic components (organic materials like collagen fibers and bone marrow cells), while approximately 80% of human teeth consist mostly of calcium [10]. Studies have shown that HAp possesses a greater ability to repair bones and cartilage compared to other biomaterials; for example, polylactic acid, polyglycolic acid, hydrogels, and nanofiber composites [11]. However, its fracture toughness and flexural strength may not be sufficient to bear the load of a skeleton, and its reliability as a synthetic bone material is sometimes poor compared to natural bones. To address these limitations, metallic implant composites like titanium-based ceramics (Ti/HAp), stainless steel-based ceramics (SS/HAp), and other metallic–HAp combinations have been introduced [12,13]. In addition to bone strength, chemical stability can also be improved. Thus, researchers focus on incorporating calcium phosphate into biopolymers to enhance chemical stability and bone strength. This approach involves mixing biopolymers with bioceramic compounds, where the biopolymer provides chemical stability and the bioceramic imparts bone strength [2,14].

Another method to create synthetic biocomposites is by coating metallic materials with hydroxyapatite or scaffolding [15]. These scaffolds can be prepared by coating materials on implants or by polymer infiltration, increasing the lifespan of synthetic biomaterials. Nanomaterials are also added to enhance specific properties of implants, such as surface features, which react favorably with the body environment. As these biomaterials integrate into the body, they create space and become favorable to the body environment, allowing bones and tissues to regenerate while the scaffolds undergo degeneration [16]. Currently, various techniques like bone grafting, tissue grafting, bone repair, regeneration, and prostheses are used for the replacement of natural bones [17,18].

This article focuses on hydroxyapatite biopolymer composites, which represent a versatile type of material with immense potential for a wide range of biomedical applications. Thus, this unique combination of biocompatibility, bioactivity, and mechanical properties makes them highly attractive for use in tissue engineering, drug delivery, and orthopedic implants. With continued research and development efforts, these composites promise to revolutionize the field of biomedical engineering and improve patient outcomes in healthcare.

## 2. Hydroxyapatite Structure

Hydroxyapatite (HAp) is classified as a highly bioactive and biocompatible inorganic calcium phosphate material naturally occurring in human bone and teeth compounds. The approval of bioactivity is determined by its ability to deliver a steady supply of chemical signals that stimulate cell function and tissue growth [19]. The most common calcium phosphate compounds and their main characteristics are illustrated in Table 1. Calcium phosphate CaPO_4_ is characterized by its ionic Ca/P ratio, basicity/acidity, and solubility, which are strongly concerned with the pH value of the solution. HAp is considered a stable calcium phosphate at normal temperatures, characterized by a Ca/P molar ratio of 1.67 and pH values ranging between 9 and 12 [8].

**Figure 1:**
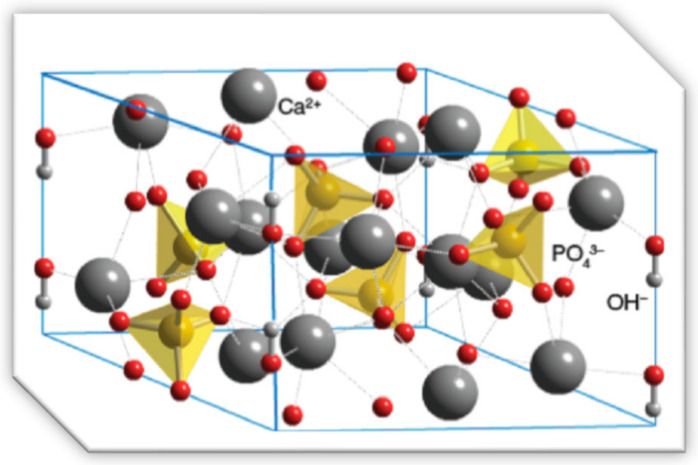
Crystalline structure of HAp [21].

Pure HAp usually crystallizes in the monoclinic space group, However, at temperatures above ~250 °C, a monoclinic-to-hexagonal solid-state phase transition is predicted. The hydroxide ions in hexagonal HAp were revealed to be more disordered inside each row when compared to the monoclinic, moving upward or downward in the structure. This promotes hexagonal lattice strains that require charge compensation by substitutions or hydroxide vacancies. Moreover, the hexagonal HAp is a popular type in biology and medicine. The hexagonal structure of HAp is illustrated in Figure 1. Hydroxide (OH^−^) can be substituted by carbonate, fluoride, or chloride to form carbonateapatite, fluorideapatite, and chlorideapatite, respectively. Bone mineral is a modified type of HAp that constitutes up to 50% of the bone volume and approximately 70% of the human bone weight. Essentially, all dental enamel and dentin contain carbonated calcium-deficient hydroxyapatite [20,21].

**Figure 2:**
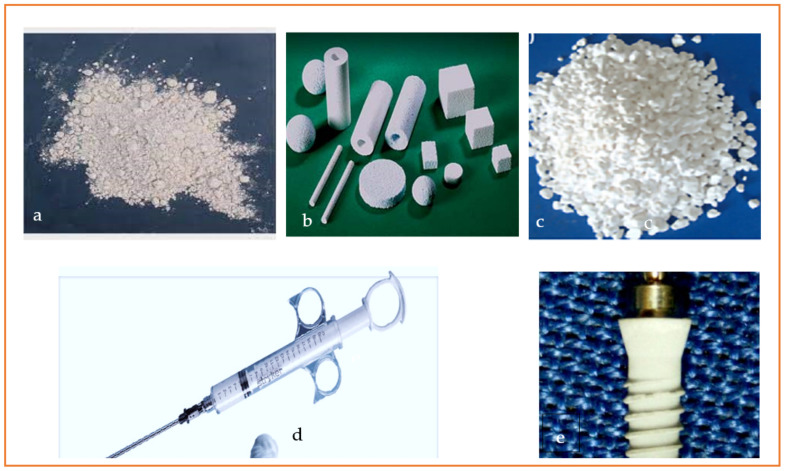
Various forms of hydroxyapatite biomaterials: (**a**) powder, (**b**) porous block, (**c**) granular, (**d**) cement, (**e**) coating [2,22]. Reproduced with permission from the *Int. J. Biolog. Macrom.*; published by Elsevier [2023].

Depending on the applicable uses, HAp is found in various forms, for example, powders or granules, for repairing bones, porous 3D scaffolds for filling bone defects, and cement forms for small surgical cuts, as shown in Figure 2 [2,22].

## 3. Synthesis of Hydroxyapatite

Because of the wide applications of hydroxyapatite in biomedical fields, such as bone tissue engineering, dental implants, drug delivery, and wound healing, researchers have been interested in synthesizing this bioactive material. However, chemically synthesized HAp poses certain drawbacks, including high costs, complexity, lengthy processing times, and the generation of undesirable by-products [23]. Moreover, chemically synthesized HAp exhibits lower biological activity in bone regeneration and delays in bone resorption processes due to the absence of essential ions. To address these challenges, different synthesis techniques have been explored, including dry methods (solid state and mechanochemical reactions), wet methods (hydrolysis, precipitation, hydrothermal, and sol–gel), and high-temperature processes (spray pyrolysis, combustion, and thermal decomposition). Hence, each of the synthesized methods results in different sizes and morphologies and yields various crystalline phases of the calcium phosphate in addition to pure crystalline HAp. Additionally, the properties of HAp significantly influence bioactivity, mechanical behavior, and biological properties [24,25].

### 3.1. Dry Methods

**Figure 3:**
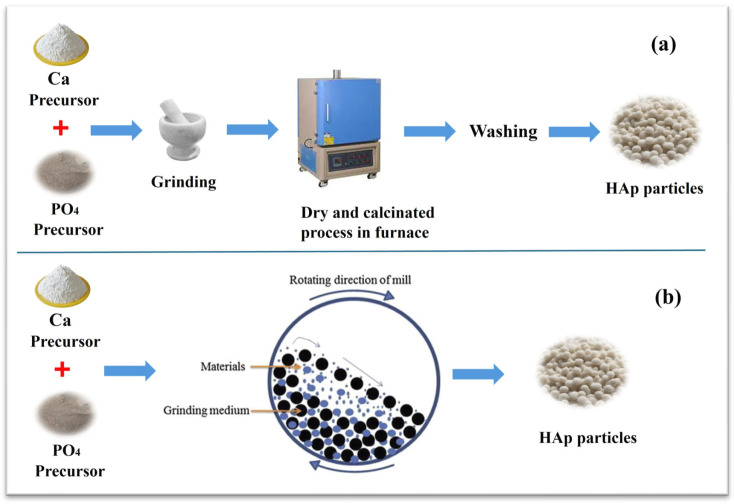
Synthesis of HAp powder via (**a**) solid state and (**b**) mechanochemical methods.

In the dry method, the initial reactants, which are in a dry form, are mixed and calcined at a high temperature to synthesize HAp. Dry methods are widely used for the mass production of powders, involving two methods: solid state and mechanochemical processes. In the solid state, precursor chemicals (calcium and phosphate) are mixed and calcined at high temperatures (≈1000 °C) within a specific time. It is a relatively low-cost and not complex procedure. While the mechanochemical process involves grinding the precursors and using mechanical energy to develop structural changes and chemical reactions, this production method simply makes powders with a well-defined structure, enabling the fabrication of different types of advanced materials [6]. A common dry method for producing HAp powder yields a product that is typically large and irregular in shape; the general process of solid state and mechanochemical processes is shown in Figure 3. Natalia et al. used a solid-phase mechanochemical synthesis to produce HAp doped with zinc or copper ions with the aid of a planetary ball mill; the synthesis took half an hour, and hydrated phosphate reagents were used to make the neutralization reaction more effective. The phase composition of the doped samples was examined, and the result revealed that dopant ions primarily occupy calcium ion sites [26].

### 3.2. Wet Methods

The wet process occurs when calcium and phosphate precursors react chemically in a solution using different solvents and temperatures. The wet method consists of a set of techniques that involve sol–gel, hydrothermal, and hydrolysis; using these methods can control the powder morphology and mean size of the particles.

In the conventional sol–gel process, precursor chemicals are mixed and turned into solid particles uniformly dispersed in a solution. The particles are then followed by gelation, drying, and calcination to remove all organic residue compounds. This preparation method is most widely used to produce nanosized HAp powder with regular morphology. Many researchers prepare hydroxyapatite using sol–gel methods, which involve hydrolyzing precursors. Yu et al. utilized trimethyl phosphate to prepare hydroxyapatite nanoparticles [27]. The solvothermal process allows for the preparation of dendritic structures of hydroxyapatite materials, which are advantageous for reactivity and regeneration [28]. Synthesis nano- and micro-sized monodisperse HAp particles can be achieved using calcium nitrate tetrahydrate and phosphorous pentoxide P_2_O_5_ [29]. The synthesis of acicular hydroxyapatite crystals in water systems, which are strengthened for biomedical materials, uses dicalcium phosphate as a precursor to form a crystal of phosphate–dicalcium phosphate–hydrate–octacalcium–phosphate [30]. Luo et al. employed the precipitation method using an ultrasonic field to synthesize nano-hydroxyapatite crystals [31]. The internal structures of these plate-like crystals contained oriented, transversely connected nanorods. Examinations of a single nanorod’s microstructural structure reveal a highly uniform, defect-free lattice structure, and a unique crystal surface orientation was observed. Thus, the obtained structure can be attributed to ultrasound’s growth mechanism [31].

**Figure 4:**
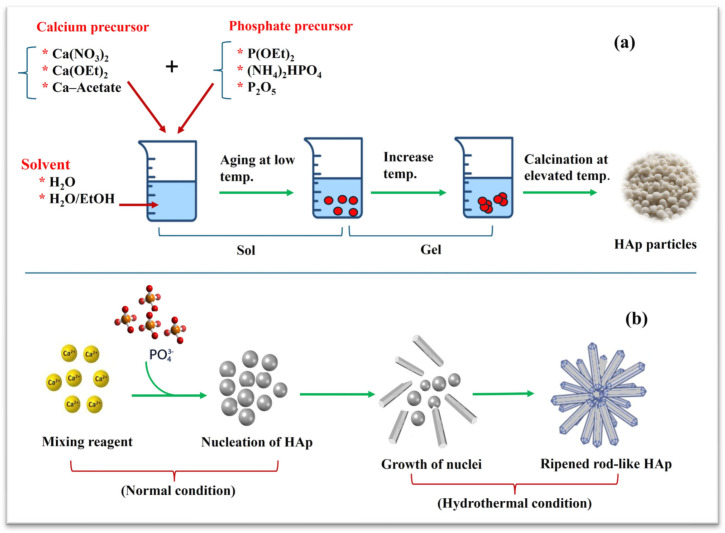
Synthesis of HAp powder via (**a**) sol–gel and (**b**) hydrothermal methods. Reproduced with permission from the *Int. J. Biolog. Macrom.*; published by Elsevier [2023].

The hydrothermal method, as high-temperature chemical precipitation, uses organic additives to control the crystals’ morphology and structure. During the preparation process, the interaction between chemicals takes place at high pressure and temperatures, resulting in a highly crystalline HAp structure in micro- or nanosize with controlled porosity depending on temperature and pressure applied. The most significant disadvantage of the hydrothermal method is the low ability to control the nanoparticle morphology and size distribution and the cost of equipment used at elevated temperatures and pressure. Figure 4 shows the schematic diagram of the steps involved in sol–gel and hydrothermal methods. Suchanek et al. achieved the acicular nature of hydroxyapatite crystals through hydrothermal preparation using calcium and ammonium phosphate raw materials [32]. Szterner et al. prepared whisker hydroxyapatite by the hydrothermal method in a reaction and showed the effect of temperature and pressure on the morphology of the products [33]. Bensala et al. produced nanorod-structured hydroxyapatite from dihydrogen phosphate and phosphogypsum waste products by hydrothermal synthesis, and HAp purity and morphology greatly depend on synthesis conditions [34]. Wen’s research group synthesized porous structures of hydroxyapatite in an ammonium phosphate solution with calcium titanate as a raw material and synthesized hexagonal hydroxyapatite acicular crystals using calcium nitrate, potassium hydroxide, potassium phosphate, and glutamic acid [35].

The hydrolysis technique known as water ionization involves the diffusion of hydrogen and hydroxide ions. Using the hydrolysis technique offers HAp nanoparticles by transforming calcium phosphate phases in an aqueous solution, and most hydrolysis methods require long processing times to complete the transformation to HAp. Mechay et al. prepared HAp nanoparticles in the polyol medium (propane diol and ethylene glycol) using the hydrolysis method. The process involves pouring a calcium nitrate drop by a drop into a diammonium phosphate solution at a temperature reaching 136 °C with a Ca/P ratio of 10:6 using a peristaltic pump for 3 h and adjusting pH to 10. An analysis of the phase and composition of polycrystalline was performed by TGA/DTA, FT-IR, TEM, and XRD, and the results indicate high crystallinity HAp nanoparticles obtained by this method [36].

### 3.3. High-Temperature Processes

**Figure 5:**
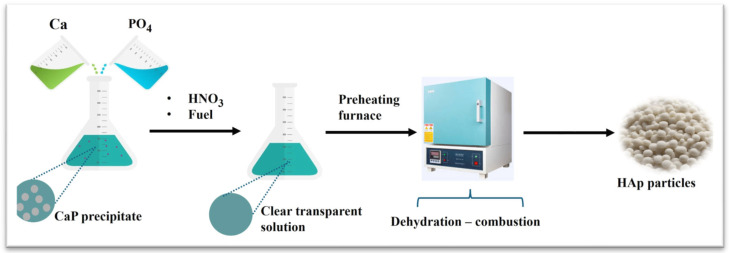
Synthesis of HAp nanoparticles via the combustion method.

In the high-temperature synthesis, the starting precursors decompose and react via elevated temperature to produce HAp. There are two main types of this method: (1) combustion and (2) pyrolysis. The combustion process enables the production of HAp ceramics in one step with high purity. It occurs between an oxidant and an organic fuel like urea, hydrazine, and glycine in an aqueous solution in a fast, low-energy, exothermic reaction. The exothermic reaction is self-propagating and does not need additional energy. Figure 5 schematically shows the steps involved in this process.

However, adjusting and monitoring processing criteria like temperature, fuel, and precursor are essential, which affects final HAp powder properties. Sasikumar and Vijayaraghavan produce HAp nanoparticles through combustion synthesis utilizing citric acid and succinic acid as fuels. Their results clearly show that carbonate HAp is gained from each type of fuel used; in contrast, β-TCP is only formed when fuels are mixed [37]. During pyrolysis synthesis, product particles are created by atomizing the starting solution and heating the droplet without fuel addition. With the aid of an ultrasonic spray generator, the precursor solution was sprayed on the blaze of an electric furnace. Vallet et al. published a study on the pyrolysis synthesis of HAp using an ultrasonic frequency. Their method included the ultrasonic frequency of a precursor solution containing calcium chloride and ammonium dihydrogen phosphate to produce an aerosol with droplets as small as 2–4 µm [38]. The emulsion synthesis technique can be accomplished by three different main categories: water in oil, oil in water, and water in oil in water. The emulsion method does not require high temperatures, and it is more effective in managing particle size, morphology, and aggregation of HAp produced [23].

## 4. Hydroxyapatite from Biowaste Materials

Utilizing biogenic residues to produce calcium phosphate compounds, particularly HAp, presents a promising avenue for cost reduction in obtaining biomaterials. Biogenic sources such as eggshells, shells, and corals offer a viable means of extracting hydroxyapatite [39].

**Figure 6:**
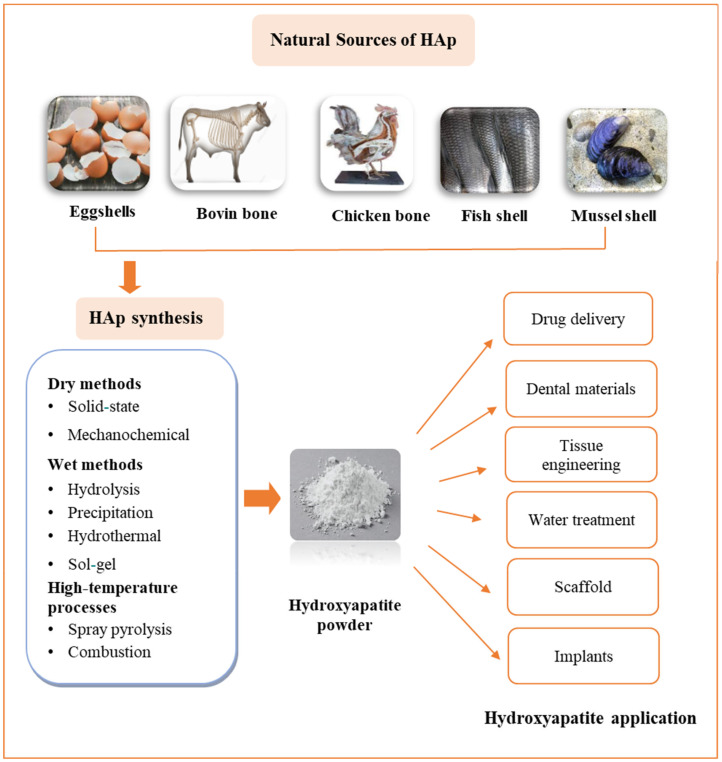
Synthesis of HAp from natural sources.

Additionally, calcium-rich materials from biogenic origins such as bovine bones, eggshells, fish bones, and scales can serve as valuable sources of hydroxyapatite (Figure 6). Notably, hen’s eggshells, fish bones, and scales stand out as compelling calcium sources due to their abundance and accessibility, thereby lowering raw material expenses and offering environmental advantages through residue utilization [18,40,41]. Inadequate disposal of these materials may foster bacterial and fungi growth due to the presence of organic matter, leading to unfavorable outcomes, such as unpleasant smells and the potential for disease transmission.

### 4.1. Eggshells

**Figure 7:**
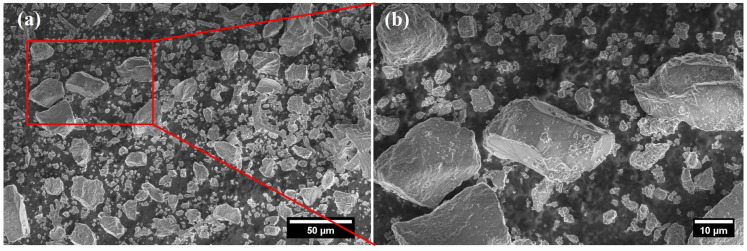
Surface morphology of eggshell powder by SEM at two different magnifications. (**a**) low magnification SEM image, and (**b**) Magnified SEM image of the region marked by a red rectangle.

Eggshells are natural bioceramic materials with a unique chemical composition of 95% crystalline calcium carbonate CaCO_3_ surrounded by a 5% protein framework. Therefore, it serves as an appropriate starting precursor of calcium for the synthesis of HAp [39]. The surface morphology of eggshell powder was investigated by scanning electron microscopy (SEM), as shown in Figure 7.

**Figure 8:**
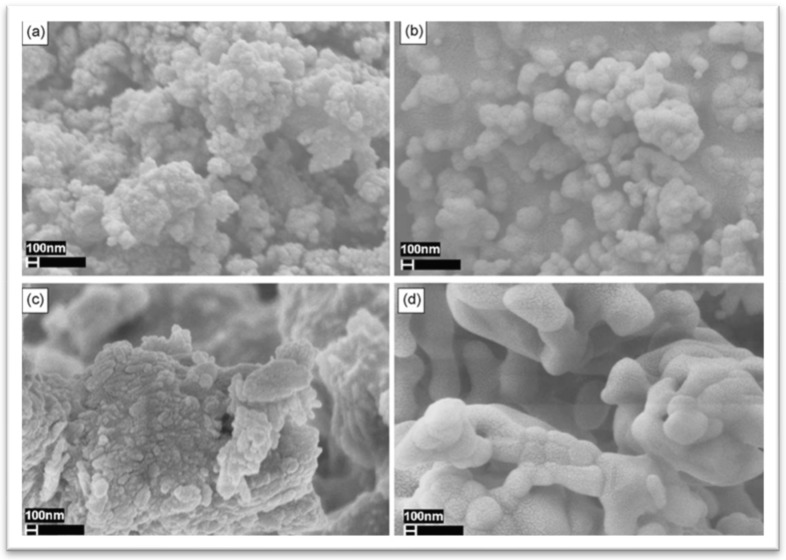
Scanning electron images of milled and attrition eggshells: (**a**) attrition milling, (**b**) attrition milling after heat treatment at 900 °C, (**c**) ball milling, (**d**) ball milling after heat treatment at 900 °C [43]. Reproduced with permission from *Ceram. Int.*; published by Elsevier [2009].

The eggshell was heat treated at various temperatures to transform CaCO_3_ into calcium oxide CaO, which was then further treated with diammonium hydrogen phosphate to obtain pure calcium phosphate. Azis Y synthesized hydroxyapatite nanoparticles from eggshells using the sol–gel method to form precipitated calcium carbonate followed by adding 0.3 M of (NH_4_)_2_HPO_4_ to increase the solubility in water at different conditions of aging time, Ca/P ratio, and pH value. The result demonstrated that the pure hexagonal structure of hydroxyapatite was achieved at 24 h aging time and pH 9 [42]. Gergely et al. prepared hydroxyapatite from recycled eggshells by thermal treatment in two stages. In the first stage, most of the organic materials were burned out, while the second stage involved the transfer of eggshells to calcium oxide, and a ball mill with different speed rotations was used to prevent agglomeration and ensure homogenous mixing of calcined. The resultant data obtained from XRD, FTIR, and SEM proved that attrition milling (4000 rpm for 5 h) is more efficient than ball milling (350 rpm for 10 h); as a result, in nanosize, homogenous Hap, even after milling, is shown in Figure 8 [43]. Sundaram et al. synthesized hydroxyapatite using nanoparticles in the presence of drug molecules, utilizing ciprofloxacin from eggshells as calcium precursors within 30 days, establishing an environmentally friendly method [44]. Castro et al. synthesized hydroxyapatite through hydrothermal eggshell synthesis via microwave irradiation methods [45]. A research study by Zhao et al. tried to use chemical treatment instead of heating steps by reacting eggshells with dilute hydrochloric acid to obtain calcium chloride [46]. Recently research by Mohd et al. prepared HAp from eggshells using chemical precipitation and calcination methods. The calcination method was performed at different temperatures (300 °C, 500 °C, 700 °C, 900 °C, and 1100 °C), and then the calcinated powder dissolved in water with 0.6 M of phosphoric acid. According to data, 700 °C was the optimum calcination temperature for HAp [47].

Lee et al. evaluated the physical properties of hydroxyapatite extract from eggshells and synthetic hydroxyapatite by FT-IR and XRD and studied the bone regeneration capability of a rabbit calvarial defect model (vivo test). The results indicated that bone formation was higher with both types of HAp than with the unfilled control [48]. Similar results were achieved by Balázsi et al. using nanosized hydroxyapatite for bone regeneration of a rabbit and mouse calvarial defect model, and the results were investigated by transmission electron microscopy (TEM) [49]. Eggshells may provide an economical source of hydroxyapatite for bone grafting. Hence, these preliminary findings suggest that the synthesized hydroxyapatite derived from hen’s eggshells traditionally considered waste has potential applications in tissue engineering.

### 4.2. Mammalian Bones

Mammalian sources such as horse, camel, pig, and bovine mainly comprise calcium carbonate (CaCO_3_) and thus represent an excellent potential for hydroxyapatite extraction. Bovine bones are widely used in the extraction of HAp rather than other mammalian sources. The bone is pretreated before extraction by washing with hot water or solvents, removing all fat, proteins, and blood cells. To ensure the removal of all organic matter, the bone is heated to 1400 °C during the synthesis process. Barakat et al. investigated the effect of three different methods on the synthesis of HAp from bovine bones by thermal decomposition, subcritical water, and alkaline hydrothermal processes. Based on analysis data, the alkaline hydrothermal method produces pure HAp nanorods compared to nanoparticles obtained from the other two techniques [50]. Odusote et al. used the thermal decomposition method to extract HAp from bovine bones after processing powder calcination at a variety of temperatures and times. The physicochemical experiments have revealed that temperature and time play a crucial role in the properties of the final product, and the results found that a temperature of 750 °C for 6 h is identical in producing pure HAp [51].

### 4.3. Marine Source

Many marine wastes like mollusk, shellfish, cuttlebone shellfish, oyster shellfish, and snail shellfish have become extensively studied sources for the synthesis of HAp, and these species are rich in CaCO_3_ content. Thermal calcination, which involves heating at elevated temperatures to remove volatile components, is the most common technique to obtain natural HAp. Zuliantoni et al. prepared hydroxyapatite from snail shells using the hydrothermal method with phosphate hydrogen diammonium. The characteristic analysis shows excellent purity of HAp made of snail shell as compared to standard HAp. Nano-hydroxyapatite can be produced from mussel shells. The shells of mussels comprise about 55% of the entire weight, and they contain 95–99% aragonite, which is a form of calcium carbonate (CaCO_3_) [41]. Liand A. et al. utilized the seafood waste of green mussel shells to achieve hydroxyapatite through calcination at high temperatures of 800 °C, 900 °C, and 1000 °C for two-hour sessions with the microwave irradiation method, which detected that the crystallin HAp phase had a significant difference among the different samples. The resultant synthesis was applied to spiro oxindole compounds [52].

## 5. Composite of Natural Biopolymer and Hydroxyapatite

Despite the frequent use of hydroxyapatite in medical applications, due to its favorable properties, like bioactivity and osteoconductive, hydroxyapatite in its nature has high brittleness and difficulty in fabricating the desired size and shape [53]. Thus, the production of biocompatible hydroxyapatite composites is under focus, as it improves the mechanical behavior of the hydroxyapatite-based implants without losing their favorable bioactive properties that are required in bone replacement engineering. Furthermore, studies have demonstrated that implants fabricated from nanosized hydroxyapatite or nanocomposites of hydroxyapatite perform better than those made from micronized ones [7].

The synthesis of hydroxyapatite biopolymer composites involves incorporating HAp nanoparticles into a biopolymer matrix through various techniques such as blending, in situ polymerization, electrospinning, and freeze drying [14]. Each method offers distinct advantages in terms of control over composite properties, scalability, and cost effectiveness. For example, blending allows for the easy incorporation of HAp nanoparticles into biopolymer solutions, while electrospinning enables the fabrication of nanofibrous scaffolds with high surface area and porosity [54]. For the fabrication of HAp-containing biocomposites in thin film form, melt compounding is proposed as an efficient method. However, to ensure that HAp is well dispersed within the polymer matrix, a solvent casting method is recommended before the materials are subjected to melt compounding via a Brabender [55].

The properties of hydroxyapatite biopolymer composites can be tailored to meet specific biomedical requirements by adjusting parameters such as HAp particle size, biopolymer composition, and processing conditions. These composites exhibit excellent biocompatibility, promoting cell adhesion, proliferation, and differentiation, making them suitable for tissue engineering applications. Moreover, the incorporation of HAp nanoparticles enhances the mechanical strength and bioactivity of biopolymer matrices, improving their suitability for load-bearing implants and drug delivery systems. The improved mechanical properties of polymer composites containing HAp as a filler can be attributed to the intermolecular interaction with the polymer chains that have a high affinity for HAp, based on a study conducted on polyurethane. The results demonstrated that the higher the amount of HAp, the more restriction to the mobility of polymer chains providing enhanced mechanical properties of polymer composites [55,56]. Alternatively, scaffolds for bone regeneration must possess specific attributes, including biocompatibility, ample mechanical strength to withstand loads, and biodegradability without producing toxins within the body. The ceramics and polymer materials comprising these scaffolds require rigorous testing to ensure their appropriateness. Key scaffold biomaterial properties should encompass (I) the availability of processing techniques utilizing accessible materials, (II) the absence of harmful by-products during degradation and integration, and (III) efficient absorption and release capabilities.

**Figure 9:**
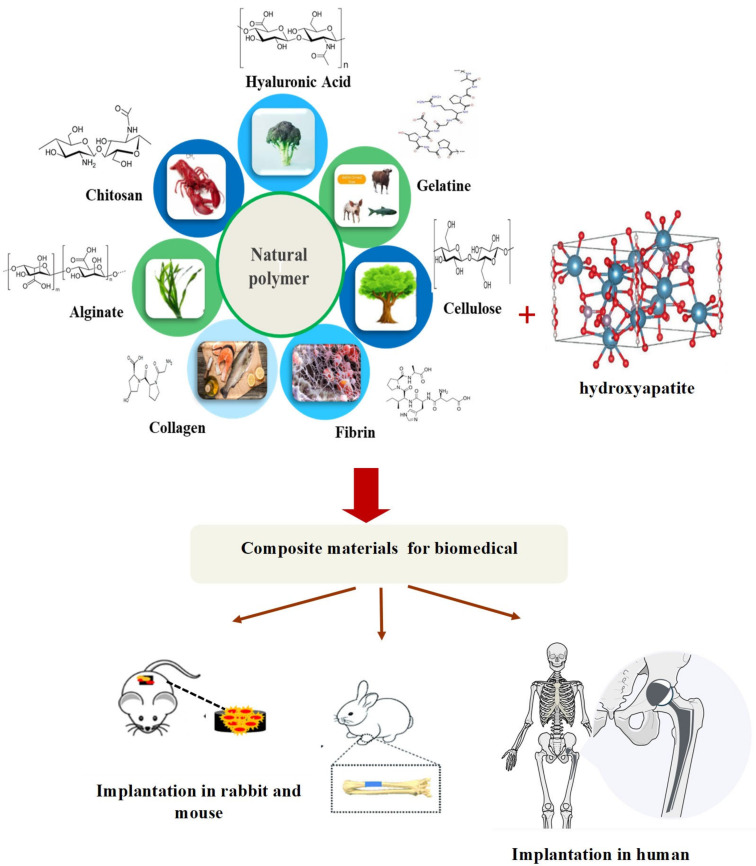
Hydroxyapatite-based natural biopolymer for biomedical application.

One promising hydroxyapatite biopolymer composite is carboxymethyl cellulose (CMC), utilized to prepare CMC/HAp composite [57]. Various methods can be employed for composite preparation, including sonochemical synthesis [58], electrospinning, and solvent casting [10]. Solvent casting involves utilizing a suitable solvent to cast the composite, while electrospinning entails adding more polymeric fibers to create denser composites. Fiber thickness increases with the amount of HAp, and the spinning process aids in binding it to the composite matrix, thereby enhancing strength. It is imperative to prevent fiber and HAp agglomeration. Some properties of the biopolymer and hydroxyapatite include excellent interfacial strength, effective reagent interaction, strong bonding between the polymer and HAp, and biopolymer degradation upon completion of its function [59] (Figure 9).

### 5.1. Collagen

**Figure 10:**
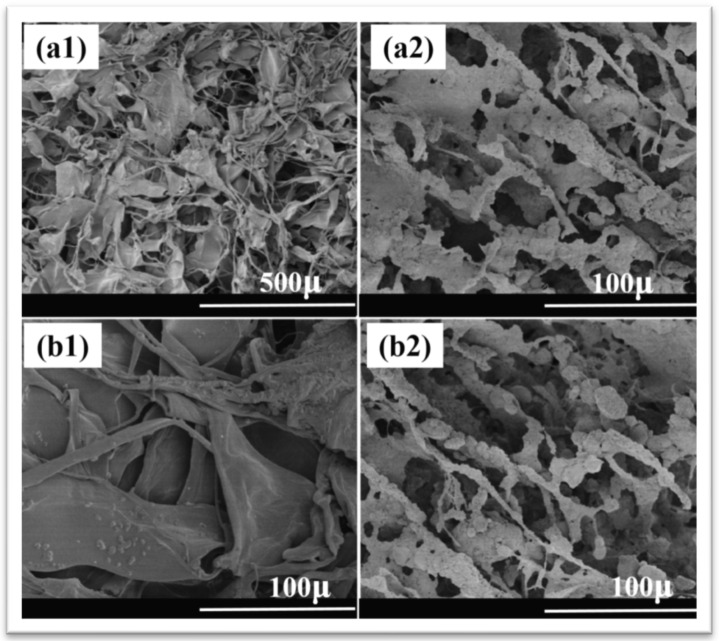
SEM images of collagen (**a1**,**a2**) at 100× and 500× and HAp/collagen composites (**b1**,**b2**) at 100× and 500× magnification [61]. Reproduced with permission from *RSC Adv.*; published by *R. Soc. Chem.* [2023].

Collagen, an essential component of the extracellular matrix (ECM) in our bodies, serves diverse functions in various ratios and forms. Bone, consisting of an inorganic–organic composite structure with hydroxyapatite nanorods embedded in a collagen lattice, undergoes nucleation and development of inorganic crystallites during biomineralization [60]. Collagen’s triple-helical structure, containing fibrils acting as nucleation sites for nano-apatite particle growth, influences cell adhesion and tissue development. Collagen/hydroxyapatite composites are extensively studied for bone tissue engineering applications. The combination of collagen’s bioactivity and hydroxyapatite’s osteoconductivity and bioactivity promotes cell adhesion, proliferation, and differentiation, leading to enhanced bone regeneration. These composites serve as scaffolds to support new bone formation and integration with the surrounding tissue. Figure 10 shows collagen’s SEM smooth-surface porous structure compared to a rough and porous structure with nano-HAp particulates adhered to the collagen surface [61].

The combination of collagen with porous HAp scaffolds bolsters their mechanical integrity by diminishing porosity [62]. This enhancement in mechanical attributes arises from the development of intermolecular hydrogen bonds between collagen and HAp, augmenting breaking energy. Moreover, nanosized HAp particles surpass their micro-sized counterparts in efficacy due to their larger surface area, facilitating accelerated and enhanced bone bonding between the scaffolds and neighboring host bone tissue containing HAp components. Researchers are actively exploring diverse methodologies to fortify the strength of Hap–collagen composites. For instance, Cunniffe et al. suggested the integration of nanosized hydroxyapatite (nHAp) particles into collagen scaffolds. Resulting in a highly porous and interconnected structure, the collagen/nHAp scaffolds exhibited a remarkable 18-fold increase in compressive modulus when 500 wt.% nHAp was added using the suspension method [63].

### 5.2. Gelatin

Gelatin composites, noted for their low antigenicity and biomaterial applicability, have garnered attention [64]. Derived from collagen hydrolysis and denaturation, gelatin is a water-soluble biopolymer with numerous ionizable groups, both naturally occurring and synthetic, offering versatility [65]. Gelatin retains collagen’s arginine–glycine–aspartic acid (RGD) sequence, promoting cell adhesion and differentiation, and is obtained from various sources such as fish, ducks, or animal bone powder. Organic and biodegradable, gelatin’s polyampholyte nature contributes to its properties, making it suitable for physiological environments. When combined with hydroxyapatite, gelatin yields a robust product ideal for tissue and cell engineering.

**Figure 11:**
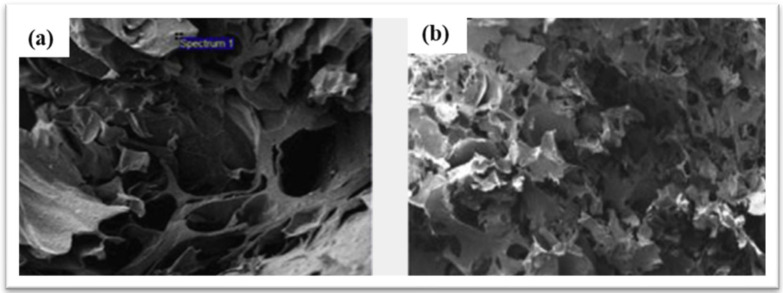
SEM-EDX spectrum image: (**a**) gelatin and (**b**) composite material of gelatin/Hap [68]. Reproduced with permission from *Heliyon*; published by Elsevier/Cell [2019].

Various concentrations of hydroxyapatite (HAp) were combined with gelatin electrospun fibers to create scaffolds for investigating the behavior of human fetal osteoblasts. At the same time, the oriented fibers have led to improved overall mechanical properties. However, the oriented gelatine nanofiber is more effective in tissue engineering scaffolds as it provides chemical cues, which are important in tissue engineering [66]. Additionally, injectable enzymatically crosslinkable gelatin and functionalized gold nanoparticles have been offered as biodegradable bone grafting, serving as suitable templates for drug and cell delivery in tissue engineering [67]. The adjustment of component proportions in gelatin composites with chito-oligosaccharides and magnesium calcium phosphate formulations was observed to regulate the pore size of scaffolds, directly influencing osteogenic differentiation [4,40]. Kim et al. focused on preparing HAp–gelatin composites, emphasizing characterizations of crystal structure and composite morphology. Their results proved the creation of a micro-porous HAp–gelatin composite scaffold, featuring interconnected pores and a micro-porous morphology of HAp particles providing elongated interfaces crucial for integration into adjacent tissues. Fourier-transform infrared (FTIR) results demonstrated chemical bonds crosslinking between the composite of gelatin and HAp particles. Furthermore, thermogravimetric analysis (TG) revealed high stability of the gelatin–HAp composite [68]. Yadav et al. analyzed and prepared HAp–gelatin composites in different ways, emphasizing crystallographic and morphological characteristics. Their findings revealed the creation of a micro-porous HAp–gelatin composite scaffold, featuring interconnected pores and a micro-porous morphology of HAp particles, as shown in Figure 11, which provided elongated interface crucialIntegration into adjacent tissues and physiological responses. According to FTIR spectra, the crosslinked composite formed chemical bonds between gelatin and HAp particles [69].

### 5.3. Chitosan

**Figure 12:**
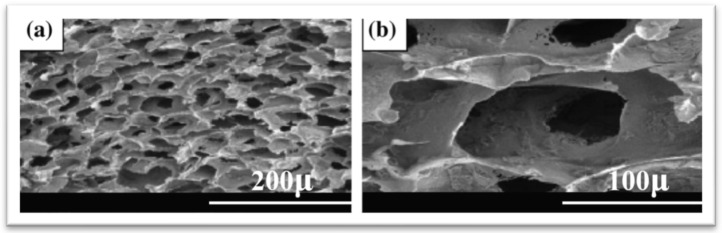
SEM of nHAp composite hybrid scaffold at different magnifications: (**a**) 200× and (**b**) 1000× [72]. Reproduced with permission from *Colloids Surf. B Biointerfaces*; published by Elsevier [2017].

Chitosan, extracted from chitin, the primary structural component of crustacean shells, undergoes deacetylation to form a polymer. It possesses a linear chemical structure containing D-glucosamine and N-acetylglucosamine in a random distribution (1–4). Chitosan can be molded into various shapes such as films, microspheres, nanoparticles, porous membranes, or scaffolds. Its characteristics, including degree and molecular weight, play a significant role in determining its properties. When combined with apatite cement, chitosan exhibits antibacterial activity [70]. This composite is formulated by blending dehydrated dicalcium phosphate and calcium hydroxide with chitosan and other additives. In recent years, there has been a notable increase in scientific articles focusing on characterizing and testing chitosan-based biomaterials. Scientists have developed various chitosan composites, such as chitosan oligosaccharide and hybrid chitosan composites, through reactions involving carbonyl compounds. Techniques utilizing cationic cellulose nanocrystals and anionic cellulose nanocrystals have been proposed for fabricating composite biomaterials, including double-membrane hydrogels. These processes typically entail two main steps: first, synthesizing organic polymeric scaffolds from chemically treated or untreated chitosan. In the second step, the scaffolds are mineralized in saturated/matrix solutions or simulated body fluid using a biomimetic method [71]. Chitosan–HAp composites are frequently combined with other biopolymeric materials. Hu et al. designed a biomimetic hybrid scaffold from hyaluronic acid, chondroitin sulfate, chitosan, and nHAp using the freeze-drying method. The results indicated improved osteoblast proliferation and differentiation and enhanced mechanical properties effectively. In addition, the structural properties of bone tissue scaffolds, including pore size and porosity, are essential for optimizing nutrient transport. The surface morphology revealed that the nanosized particle was distributed uniformly without agglomeration and a homogeneously interconnected microstructure of the composite, as shown in Figure 12 [72].

Functionalized chitosan–collagen–HAp composite scaffolds produced by lyophilization exhibited low cytotoxicity, high bioactivity, and high biocompatibility in vitro, as reported by Türk et al. [73]. In another study by Shi et al., a gradient scaffold was produced using dopamine-modified alginate, HAp, and chitosan, which exhibited low cytotoxicity and excellent osteogenic activity in vitro, promoting effective bone regeneration and accelerating bone defect repair in vivo. [74]. Similarly, chitosan was integrated into HAp scaffolds with alginate in the study by Liu et al. targeting bone regeneration applications [75]. Rahmani F. et al. investigated the ideal local conditions for the nucleation and growth of nHAp on CS films using a method based on phosphorylation, partial hydrolysis, Ca(OH)_2_ treatment, and an artificial saliva solution immersion at room temperature. The study findings were evaluated by SEM/EDX, XRD, XPS, and FTIR spectroscopy, revealing the growth of carbonate HAp crystals through the biomimetic method. Additionally, the obtained CS films have exhibited a concentration-dependent antimicrobial activity, achieving the study aims [76].

### 5.4. Fibrin

Fibrin serves as a common biological sealant for bone sealing in the final stages of grafting fibrin adhesives and sealants. Comprising fibrinogen, thrombin, calcium chloride, proteins, and antifibrinolytic agents, fibrin proves valuable for bridging bone defects and facilitating bone repair. It aids in bone proliferation, cell growth toward bone attachments, and other cellular functions [77,78]. Commercially available fibrin sealants are utilized for bone repair. However, increasing the concentration of gels with fibrins and thrombin was found ineffective, as it burdened patients with the weight of these protein-rich clothes [79,80]. Fibrin gels with enhanced mechanical strength, superior morphological characteristics, and osteogenic promotion capacities are employed with fibrinogen and sodium chloride solutions [81,82]. They hold the potential for intraoperative surgeries, major or minor operations to reduce blood loss, and post-operative patient recovery. Additionally, they can substitute tissue morphologies in the body. Collagen and fibrins are recognized as composites suitable for mechanical and strain environments within the body [83].

### 5.5. Cellulose

**Figure 13:**
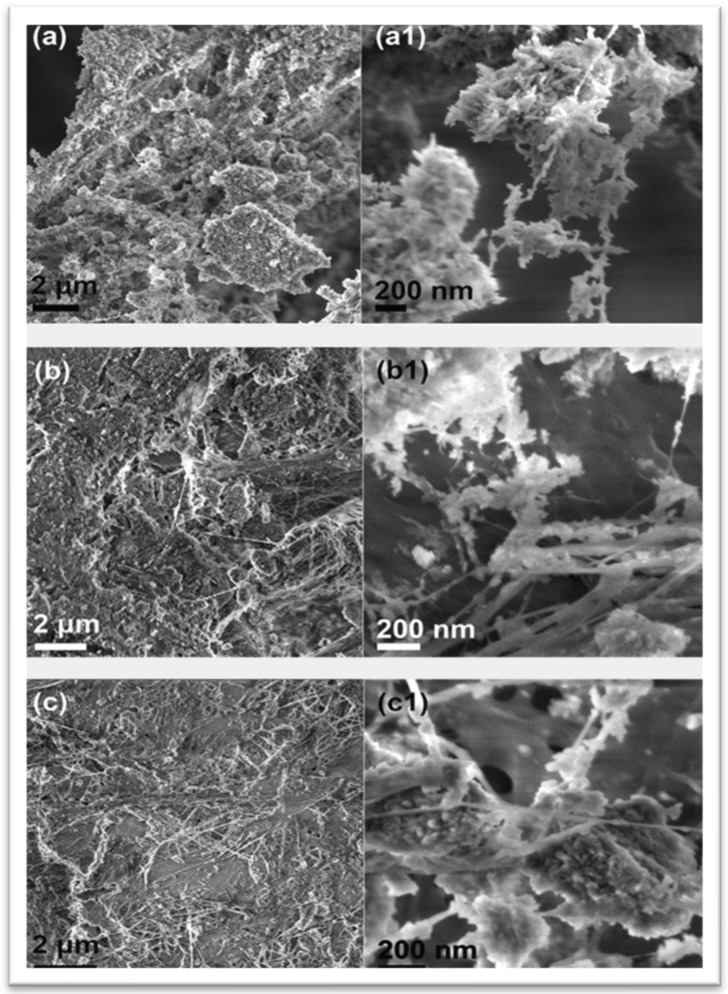
SEM images of BC/HAp composite with different content components: (**a**,**a1**) 1BC:25HAp, (**b**,**b1**) 1BC:4HAp, (**c**,**c1**) 1BC:1Hap [90]. Reproduced with permission from *Scanning*; published by Wiley [2016].

Cellulose, an abundant linear polysaccharide, is sourced from various natural sources, including cotton, bast, wood, bamboo, bacteria, fungi, and algae [84]. Original or chemically modified cellulose is commonly used for bone substitute production due to its remarkable properties, including high specific mechanical properties, non-immunogenicity, non-toxicity, abundance, and low production cost [85]. Cellulose derivatives such as cellulose acetate, cellulose esters, methylcellulose, ethyl cellulose, and carboxymethyl cellulose are extensively employed in biomaterial engineering, biomedicine, tissue engineering, scaffolds, and pharmaceutical industries. A novel approach involving bacterial cellulose microfibrils grafted onto hydroxyapatite composite demonstrates high absorption capacities compared to metal grafts, with rapid regeneration [86]. Another method utilizing cellulose scaffolds involves intermixing nano- and micro-hydroxyapatite followed by freeze drying, offering potential benefits for repetitive bone applications [87]. Tabaght et al. synthesized a biocompatible HAp/cellulose composite using a newly developed dissolution and precipitation technique that is suitable for bone replacement. In general, the co-precipitation method is crucial for the preparation of HAp–cellulose composites [88]. Sivasankari et al. studied a chemical precipitation process to synthesize HAp in cellulose acetate–polyetherimide composites. In this study, they proposed an easy and affordable method for producing materials for use in applications, such as adsorption membranes or biomedical objects. The experimental investigations demonstrated that the presence of HAp nanoparticles improves the thermal and hydrophilic properties of the composite [89]. Arkharova et al. proposed a novel method of using HAp nanocrystals to prepare and investigate the physical properties of bacterial cellulose (BC)/HAp composite with different ratios of constituents to obtain optimum products with a controlled structure and optimized properties, like natural bone. The results indicated that the final properties of the obtained composite are highly dependent on the varying ratio of BC to HAp. An increase in cellulose content leads to a decrease in HAp crystal length, porosity, and pore size, while density, tensile strength, and Young’s modulus increase. SEM analysis demonstrated that the BC fiber fragmented with attached and aggregated HAp nanoparticles. The HAp crystal warps the surfaces of BC fibers, as seen in Figure 13 [90].

### 5.6. Alginate

Alginate, a biopolymer, forms bonds with chloride ions (Na^+^) or calcium ions (Ca^2+^) to create a reticulate structure. These properties make biopolymers highly versatile across various applications, including water treatment, packaging materials, textiles, agriculture, pharmaceuticals, electronics, and biomedicine [91]. Alginate can be shaped into diverse forms such as foams, microcapsules, gels, fibers, and matrices, increasing the diversity of alginate composites with HAp. Composites combining gelatin with alginate and other polymers have been developed [92]. Combining alginate with hydroxyapatite harnesses the strengths of both materials, resulting in composite scaffolds with augmented properties for tissue engineering and regenerative medicine applications [93]. Alginate and hydroxyapatite represent versatile biomaterials with unique characteristics that are valuable in numerous biomedical applications. Their synergistic effects when combined can produce composite scaffolds with enhanced mechanical, biological, and therapeutic properties, making them promising contenders for tissue engineering and regenerative medicine. Porous crosslinked scaffolds containing calcium phosphate were also produced by a combination of alginate and fibrin, as demonstrated by [94]. The produced scaffolds were slowly biodegradable and exhibited adhesion properties.

Patil et al. used wet chemical precipitation and freeze-drying processes to prepare 3D porous scaffolds of Hap, which was coated with alginate–chitosan. The scaffolds that were produced had pores ranging from 30 to 280 μm and decreasing with increasing HAp content, while their mechanical strength increased proportionally [95]. Kohli et al. produced porous, crosslinked, slowly biodegradable calcium phosphate scaffolds by combining alginate with fibrin. These scaffolds demonstrated adhesion, proliferation, migration, and differentiation along the osteogenic pathway over the culture period. Alginate–HAp scaffolds have exhibited favorable physicochemical and rheological properties, as well as excellent biocompatibility, with cell growth and proliferation rates suitable for clinical applications [94]. In brief conclusion, alginate–HAp is a promising scaffold material, as it has excellent biocompatibility, and cell growth is favorable for applications, like clinical applications. Additionally, this combination has physicochemical and rheological properties, making it suitable for tissue engineering. However, further investigations are necessary to optimize its physicochemical properties to enhance its suitability for tissue engineering applications in bone regeneration.

### 5.7. Hyaluronic Acid

Hyaluronic acid, a crucial component of the extracellular matrix, has been extensively utilized in fields of craniofacial bone regeneration. Composite scaffolds immersed in hyaluronic acid have demonstrated great potential in enhancing osteogenesis and mineralization. Hyaluronic acid derivatives play an essential role in stabilizing the extracellular matrix, effectively improving osteointegration immobilizing implant surfaces [94].

Kaczmarek et al. developed scaffolds based on hyaluronic acid, chitosan, and collagen boosted with nHAp by lyophilization, confirming their biocompatibility. In vitro cell culture studies showed improved cell attachment and growth on the scaffolds enhanced with hyaluronic acid, while in vivo models on tissues surrounding the scaffolds six months after implantation generally revealed good wound healing and anti-inflammation caused by the implants. The incorporation of nHAp into the hyaluronic acid/chitosan/collagen scaffolds slowed the implant biodegradation process, resulting in a scaffold with more stable fixation contact with surrounding tissues [96].

Sujana et al. used the electrospinning method to generate biocompatible nanofibers of hyaluronic acid, poly(L-lactic acid)-co-poly(ε-caprolactone), fibroin, and HAp to mimic the native extracellular matrix. The nanofibrous scaffolds displayed greater porosity than microfibrous scaffolds, facilitating the optimal exchange of nutrients and metabolic waste [96]. Yang et al. developed a new combination for bone defect regeneration using an injectable hyaluronic acid–alginate hydrogel system embedded in exosomes, nanovesicles naturally secreted by cells [97].

## 6. Conclusions and Future Prospectives

Hydroxyapatite is considered one of the most promising candidates for synthetic human bone production because of its similarities with bone mineral components. In past decade, there has been increasing demand for using this low-cost, abundant, biodegradable, and biocompatible method for tissue engineering and drug delivery applications. However, the brittleness structure of HAp limits its application when the bone defect to be repaired is in a constantly tensioned area. Thus, considerable attention has been directed to creating HAp composites, incorporating multiple substances that can enhance their strength, weight, and stiffness, as well as adherent and cell growth. These improvements assist in applicability and efficiency in bone repair. Hydroxyapatite can be obtained from different sources, using cost-effective methods and utilizing both organic and inorganic materials, whether natural or synthetic. This review summarizes multiple processing methods for manufacturing hydroxyapatite composites from different naturally occurring biological sources.

Hydroxyapatite nanoparticles hold great potential as a replacement for base composites found in the human body. Hydroxyapatite-based scaffolds can be developed by employing conventional fabrication approaches, including electrospinning and freeze drying, or additive manufacturing methods, such as 3D printing.

Electrospinning technology involves the formation of nano- and microstructured fibers with unique characteristics such as lightweight, a large surface area-to-volume ratio, and exceptional porosity. These properties resulted in the electrospinning method emerging as a promising process for synthetic bone analogs with biomimetic structures. Some examples of natural scaffolds include electrospun gelatin nanofibers blended with different proportions of hydroxyapatite using a solvent or water; the interaction between gelatin and hydroxyapatite prevents participation of the inorganic phase and expresses a uniform distribution of nanofibers scaffold. Other examples of natural scaffolds are collagen–hydroxyapatite and chitosan–hydroxyapatite. In addition to natural polymers, various synthetic polymers, such as polycaprolactone and polyvinylpyrrolidone, have been introduced to fabricate nanofibers by electrospinning. A common issue with these polymers is their hydrophobic nature, which restricts the homogenization and distribution of the inorganic phase, dissimilar to natural polymers that exhibit hydrophilic tendencies due to the presence of hydroxyl groups on their polymer chain.

One of the main challenges of bone tissue engineering is the lack of vascularization. Therefore, future studies aim to develop new strategies to render scaffold platforms that support angiogenesis and vascularization and focus on producing new composite nanofiber scaffolds using an electrospinning technique that offers desirable biological functionalities and mechanical properties for bone regeneration. Computational simulations can be used as an effective tool to create efficient designs of scaffolds that meet the requirements of successful bone tissue regeneration [98]. Such models can be crucial for addressing bone-related challenges.

In conclusion, hydroxyapatite-based composite scaffolds are promising candidates for repairing missing or damaged tissue and fastening the healing processes. Despite encouraging results obtained from hydroxyapatite-based composites in vitro and some in vivo studies, obstacles in the translation of biomedical research into innovation remain a big challenge. Limitations are related to the inaccuracy of preclinical models, infection, cost, and time consumption. It is essential to use animal models for accurate assessment and to make sure that animal data match what is observed in humans before clinical trials. Additionally, one important aspect that deserves consideration is the evaluation of the performance and interaction behavior of the scaffold bone tissue engineering in the defect area.

## Figures and Tables

**Table 1:** Calcium phosphate family and their major properties [20]. Reproduced with permission from Prog. Biomater.; published by SpringerNature [2016] and https://creativecommons.org/licenses/by/4.0/ (accessed on 4 January 2024).

Compound Name	Chemical Formula	Ca/P Ratio	Solubility at 25 °C (g/L)	pH Stability Range at 25 °C
Monocalcium phosphate anhydrate (MCP or MCPA)	Ca(H_2_PO_4_)	0.5	~18	0.0–2.0
Monocalcium phosphate monohydrate (MCPM)	Ca(H_2_PO_4_)_2_⋅H_2_O	0.5	~17	a
Dicalcium phosphate dihydrate (DCPD), mineral brushite	CaHPO_4_2H_2_O	1.0	~0.0088	2.0–6.0
Dicalcium phosphate anhydrous (DCPA or DCP), mineral monetite	CaHPO_4_	1.0	~0.048	a
Octacalcium phosphate (OCP)	Ca_8_(HPO_4_)_2_(PO_4_)_4_5H_2_O	1.33	~0.0081	5.5–7.0
a-Tricalcium phosphate (a-TCP)	α-Ca_3_(PO_4_)_2_	1.5	~0.0025	c
b-Tricalcium phosphate (b-TCP)	β-Ca_3_(PO_4_)_2_	1.5	~0.0005	c
Amorphous calcium phosphates (ACPs)	Ca_x_H_y_(PO_4_)_z_⋅nH_2_O, *n* = 3–4.5, 15–20% H_2_O	1.2–2.2	b	~5–12
Calcium-deficient hydroxyapatite (CDHA)	Ca_10−x_(HPO_4_)_x_(PO_4_)_6−x_ (OH)_2−x_ (0 ˂ x ˂ 2)	1.5–1.67	~0.0094	6.5–9.52
Hydroxyapatite (HA, HAp)	Ca10(PO_4_)_6_(OH)_2_	1.67	~0.0003	9.5–12
Fluorapatite (FA or FAp)	Ca10(PO_4_)_6_F_2_	1.67	~0.0002	7–12
Tetracalcium phosphate (TTCP or TCP)	Ca4(PO_4_)_2_O	2.0	~0.0007	c

a Steady at temperatures greater than 100 °C. b Unable to be measured precisely. c Unable to be precipitated from aqueous solutions.
